# Translation, cultural adaptation, and validation of the PHQ-9 and GAD-7 in Kinyarwanda for primary care in the United States

**DOI:** 10.1371/journal.pone.0302953

**Published:** 2024-10-17

**Authors:** Frank Müller, Alexis Hansen, Megan Kube, Judith E. Arnetz, Omayma Alshaarawy, Eric D. Achtyes, Harland T. Holman

**Affiliations:** 1 Department of Family Medicine, College of Human Medicine, Michigan State University, Grand Rapids, Michigan, United States of America; 2 Corewell Health Family Medicine Residency Clinic, Grand Rapids, Michigan, United States of America; 3 Department of General Practice, University Medical Center Göttingen, Göttingen, Germany; 4 Graduate Medical Education in Psychiatry, Trinity Health Livonia Hospital, Livonia, Michigan, United States of America; 5 Department of Family Medicine, College of Human Medicine, Michigan State University, East Lansing, Michigan, United States of America; 6 Department of Psychiatry, Western Michigan University Homer Stryker M.D. School of Medicine, Kalamazoo, Michigan, United States of America; University of Malaga: Universidad de Malaga, SPAIN

## Abstract

**Background:**

Depression and anxiety are significant health burdens that greatly impact the quality of life of refugees and migrants. In this study, we have translated and culturally adapted the Patient Health Questionnaire (PHQ-9) and Generalized Anxiety Disorder Screener (GAD-7) into Kinyarwanda and performed a validation study in a United States (US) primary care setting.

**Methods:**

A committee of seven experts including psychiatric and family medicine providers, health researchers, and trained medical interpreters translated and culturally adapted the PHQ-9 and GAD-7, and incorporated feedback from cognitive interviews with bilingual participants. The translated instruments were then tested in a cross-sectional validation study. Analyses include internal consistency, discriminant validity, principal component analyses, and confirmatory factor analyses.

**Results:**

Analyses of 119 responses indicated overall good internal consistency with Cronbach’s α of 0.85 (PHQ-9) and 0.92 (GAD-7). Both scales showed acceptable factor loadings between 0.44 and 0.90 in the principal component analyses and showed strong correlations with health-related quality of life and depression/anxiety symptoms measured with visual analog scales. Significantly higher scores for PHQ-9 and GAD-7 were shown among participants with known psychiatric conditions.

**Discussion:**

PHQ-9 and GAD-7 demonstrated commendable applicability for Kinyarwanda-speaking patients in primary healthcare settings in the US. Our instruments can already be used in primary care settings and thus help to mitigate health disparities. Future research should further validate our tool against gold-standard diagnostics in larger, geographically diverse samples.

## 1. Introduction

Depression and anxiety are major global health burdens, affecting approximately 322 million and 264 million people worldwide, respectively [1]. Both conditions can lead to disability [2], mortality [3], reduced quality of life [4], and worsening of conditions like diabetes, hypertension, or heart failure [5–7]. The United States (US) Preventative Services Task Force (USPSTF) has recommended depression screening in primary care for all adults since 2016 [8] and recently expanded this guideline for anxiety screening [9].

Refugee populations, including refugees from Rwanda [10], are particularly affected by mental health problems such as depression, anxiety, post-traumatic stress disorder, and other mental conditions [11–13]. Refugees and migrants often face mental health challenges due to stress exposure to pre-migration experiences such as violence or persecution in their countries of origins, as well as post-migration difficulties including acculturation, language barriers, social isolation, and economic struggles [14]. Also, traumatizing experiences during the migration pathway, such as arduous flights, lack of sanitation, uncertainty during asylum processes, violence and/or human trafficking can have a considerable impact on migrants’ health [15].

Western Michigan, including the metropolitan area of Grand Rapids, has become one of the main destinations for refugees and migrants from Rwanda, Uganda, and the Republic of Congo in the US forming a growing community of Kinyarwanda-speaking residents [16]. Research has demonstrated that these populations are often both highly traumatized and less likely to seek mental healthcare [17], further emphasizing the need for high-quality, culturally competent mental health screening.

Validating mental health screening tools for specific linguistic and cultural contexts is crucial to ensure accurate and effective screening, enable informed clinical decisions, and facilitate standardized assessment across different healthcare settings [18]. This is particularly important for vulnerable populations such as refugees and migrants, where culturally and linguistically adapted tools can help reduce misdiagnosis and improve patient engagement in mental health care.

The Patient Health Questionnaire (PHQ-9) is one of the most frequently used mental health screening instruments [19]. While previous research provided a Kinyarwanda translation of the PHQ-9 for use among patients with epilepsy in Rwanda [20], there were concerns regarding potential misunderstandings due to dialectal variations in Kinyarwanda and differences in healthcare contexts between Rwanda and US primary care. Hence, a new comprehensive translation and validation approach, involving cognitive interviews and close collaboration with local Kinyarwanda interpreters, was deemed necessary for our specific study population of Kinyarwanda-speaking refugees and migrants in West Michigan. Furthermore, a tool to screen for clinically relevant anxiety symptoms such as the Generalized Anxiety Disorder Screener (GAD-7) [21] has not been translated and validated in Kinyarwanda.

To address this gap in care, the objectives of this work were to translate and culturally adapt the PHQ-9 and GAD-7 into Kinyarwanda and validate both screeners in a cross-sectional study conducted in primary care clinics in Western Michigan. By publishing these validated instruments as a free resource for clinicians and researchers, we hope to contribute to the efforts to mitigate health disparities among refugee and migrant populations.

## 2. Methods

This study was comprised of two phases: In the first phase, the instruments were translated and culturally adapted using a committee framework approach including cognitive interviews with bilingual participants. In the second phase, a cross-sectional validation study was conducted in primary care clinics in Western Michigan. Results of this cross-sectional part are reported using STROBE guidelines [22]. The completed STROBE checklist as provided as S3 Appendix.

### 2.1. Translation and cultural adaptation

#### 2.1.1. Approach and translation objectives

We used the committee-based translation and adaptation framework described by Valdez et al. which includes rigorous steps of (1) planning, (2) parallel translations, (3) review, (4) adjudication, (5) pilot-testing, (6) revision, and (7) documentation, that allowed us to iterate phases as necessary [23]. We further extended this framework by conducting cognitive interviews instead of pilot-testing with bilingual participants and further added an independent back translation as a final quality control before conducting the validation study.

For the translation process, the English versions of the GAD-7 and PHQ-9 were used as sources and subsequently translated and adapted into Kinyarwanda. Kinyarwanda is a Bantu language with different dialectical variants spoken in Rwanda and also in parts of Burundi, the Democratic Republic of the Congo, Uganda, and Tanzania. The aim for the translation and adaptation process agreed upon among all authors was to generate a semantically similar version retaining format, scales, range of response options, and stimulus questions while using plain and familiar language allowing the instruments to be understood by people speaking different dialectal variants and from a diverse educational background.

#### 2.1.2. Committee members and roles

The committee-based approach to translation and cultural adaptation uses a continuous feedback and review process within a team of language, culture, and subject matter experts [24]. Our committee consisted of two clinicians (EDA, HTH) trained in family medicine and psychiatry and proficient in the provision of mental health care to refugee patients, a clinician-researcher (FM) additionally trained in psychosomatic medicine providing both clinical and research expertise, one psychiatry resident physician (MK), and one medical student (AH) to provide perspectives from different levels of training, and two trained medical Kinyarwanda interpreters who have been working for the healthcare system as on-site medical interpreters for several years as linguistical and cultural experts. Interpreters were of male and female sex, middle-aged, and both grew up in Rwanda and the US and thus were proficient in both Kinyarwanda and the English language.

The process of translation and cultural adaptation was completed in six teleconference meetings using Zoom video conferencing software (Zoom Video Communications, Inc., San Jose, CA, USA), each lasting over 60 minutes between April and June 2022.

#### 2.1.3. Planning

In the planning step, we first conducted a literature review on existing healthcare survey instruments in the Kinyarwanda language to gain insights about potential obstacles that others faced during the translation process. Furthermore, these collected instruments served as a reference in the committee discussions. During the first meeting, the process of translation and cultural adaptation was discussed. The PHQ-9 and GAD-7 were introduced, and the clinicians described how these instruments were used and results interpreted in everyday practice. Furthermore, the interpreters provided insights into the cultural perception of mental health problems among the Kinyarwanda-speaking refugee community in the US and the relevant impact on the translation and adaptation process. They highlighted that mental health problems are often stigmatized among Kinyarwanda communities, and they expressed their fear that the use of stigmatized expressions would lead to biased responses and should thus be avoided. The interpreters furthermore shared that many in the community feared being labeled as “crazy” or “mad” and were afraid that a suspicious test result would implicate that their “children will be taken away”.

#### 2.1.4. Parallel translations

After this first meeting, both interpreters independently translated the instruments within two weeks (parallel forward translation). Additionally, clinicians provided paraphrases and elaborations independently on the meaning of each item, e.g. “This item is designed to address the idea of anhedonia, not enjoying things one previously used to enjoy”. The results of these first interpretations were compiled in a Microsoft Excel (Microsoft Corp., Redmond, WA) document together with the PHQ-9 version used in the study of Sebera et al. [20] as preparation for the following committee review.

#### 2.1.5. Review

After parallel translations were completed, the review process involved the entire team discussing and refining each item to ensure both linguistic accuracy and cultural appropriateness. In the four review meetings, each item was first introduced by the clinicians and explained what role it plays in patients with clinically relevant depression or anxiety symptoms. The interpreters then discussed matching phrasing to concepts and meanings as well as the format and distinction of questions and responses. Especially challenging was the translation of colloquial metaphors in the English version, such as “feeling on edge” and finding expressions that avoid stigma. Here it became apparent, that even basic wording like “mental health” itself would imply “madness” or “craziness” in a direct translation. In the following meetings, the items were further refined and compiled into a first draft, consisting of four pages.

#### 2.1.6. Adjudication

Following the review, a separate adjudication was performed by critically examining the items for consistency, clarity, and cultural appropriateness. While most items were signed off for pilot testing some items needed further revisions and modifications of the translation, particularly focusing on nuanced cultural interpretations and potential linguistic ambiguities. Interpreters also checked for contingency, grammar, and spelling and the preliminary version of the entire survey for pilot testing was compiled.

#### 2.1.7. Pilot

The pilot testing was conducted as cognitive interviews with n = 5 bilingual English/Kinyarwanda participants (3 male, 2 female), that we recruited through the Michigan State University Department of African Language. All participants were faculty or students of various academic backgrounds located in the US and the Democratic Republic of the Congo. Cognitive interviews were carried out and video- and audio-recorded using Zoom teleconferencing software. The cognitive interviews were meant to assess the cognitive process in answering questions, e.g. if the items were understood (comprehension), and if necessary and relevant information and underlying concepts were retrieved (retrieval). Furthermore, we aimed to gain insights into the process of answer preparation of participants (judgment) and if challenges arose from formatting the answers (response) [25]. The pilot testing thus did not only focus on the survey items themselves but also the handling of the survey as a whole. Participants were first introduced to the purpose of the questionnaire and that it is administered as a standard screening instrument in a family medicine clinic during every patient visit. Then participants were asked to read out loud each item, answer it, then describe their thought process that led them to their final answer [26]. After completing all items, participants were asked to comment if any items were complex, hard to understand, perceived as odd or disturbing, or contained grammatical or spelling errors. The recorded video of each interview was reviewed by the study team and concerns raised by interviewees were summarized.

#### 2.1.8. Final revisions and documentation

A certified external interpreter who was not involved in the research project provided back-translation of both survey instruments. The notes from the cognitive interviews and the results from the back-translation were then discussed in a committee meeting to finalize the interview. The final versions of the translated instruments are included in this manuscript as a (S1 Appendix).

### 2.2. Participant recruitment and setting

Western Michigan, particularly the Grand Rapids area, has become a significant resettlement location for refugees and migrants from Central Africa, including Rwanda and the Democratic Republic of Congo over the past two decades [16]. Approximately 8,000 refugees from Congo are residing in Grand Rapids and 11 Congolese church congregations are located in the area [16]. Many of these refugees speak Kinyarwanda, as it is widely used in both Rwanda and parts of Congo.

Our study was conducted at Corewell Health, a large non-profit managed healthcare organization with 22 hospitals and more than 300 outpatient facilities in Michigan. We employed a convenience sampling method for this study. No randomization was used due to the specific nature of our target population and the clinical setting.

Participants were enrolled in two ways: First, three family medicine clinics in Western Michigan that are established in providing care to the Kinyarwanda community enrolled patients in their clinics during their routine clinic visits. Second, a Kinyarwanda interpreter at the healthcare system was briefed to enroll patients in all other primary care clinics in the health system. Results of the survey were reported to the provider with the notice that the information obtained was based on a not-yet-validated scale and therefore typical thresholds of the screening instruments may not apply. Recruitment took place from 6 June 2022 to 14 August 2023.

Patients were eligible to participate, if (a) their preferred language for the encounter was Kinyarwanda and a language interpreter was needed, (b) patients were aged 18 or older, and (c) patients agreed to participate. Patients who did not meet the inclusion criteria (e.g., those under 18, or those not requiring Kinyarwanda interpretation) were not approached for the study. Patients were excluded from enrollment when they were not able to provide informed consent.

Patients who met the eligibility criteria were approached by clinic staff or interpreters at the participating clinics. The study was explained to them in Kinyarwanda through on-site or video interpreters, and they were invited to participate. Those interested received a study information document in Kinyarwanda that included information about the study’s objectives, procedures, the voluntary nature of participation, and the anonymity of collected data. For participants with limited literacy, this information was read aloud by a Kinyarwanda interpreter. Those who agreed to participate provided written consent. Patients did not receive compensation for participating in this study.

### 2.3. Measures

Besides the two translated instruments PHQ-9 and GAD-7, the survey comprised questions on participants’ sociodemographic characteristics (gender, age, education, timing of entrance into the US, self-perceived English language proficiency) as well as if the patient has ever been told by a health care provider that they have a mental health problem (such as depression, anxiety, schizophrenia, bipolar disorder, obsessive/compulsive disorder, a substance use disorder, or other mental health problems). These items were translated in the same process as described above. As illness concepts such as bipolar disorder do not have a direct translation in the Kinyarwanda language, we added a short description highlighting the main symptoms.

Furthermore, the survey contained the Kinyarwanda version of the EQ5D-VAS to measure patients self-rated overall health [27] on a scale from 0 (“worst health you can imagine”) to 100 (“best health you can imagine”). Although the Kinyarwanda EQ5D-VAS was thoroughly translated and provided as an official resource through the EuroQol group, we could not find any literature that these instruments have been previously validated in Kinyarwanda. To broadly assess depression and anxiety symptoms in our sample and to compare them to PHQ-9 and GAD-7 items, we added further visual analog scales with the questions “Overall in the last 30 days, how depressed have you felt?” and “Overall in the last 30 days, how anxious have you felt?”. Similar scales have been used and validated in other settings [28].

Besides these numerical measures, we also checked each completed survey for annotations made by the respondents.

### 2.4. Sample size

For validation studies, typically an item-to-response ratio from 1:5 to 1:10 is favored [29], suggesting we needed at least 90 responses for the PHQ-9 and 70 responses for the PHQ-7. Our analyzed sample consisted of n = 119 responses, exceeding this minimum threshold.

### 2.5. Statistical analysis

Descriptive statistics including frequencies, percentages, means and standard deviations (SD) were used to characterize our sample and the survey responses. Internal consistency of both scales was assessed with Cronbach’s α and McDonald’s Ω. Sensitivity analyses for consistency were conducted among various sociodemographic subgroups. For each item of both scales, inter-item and inter-scale correlations were calculated.

After assessing Kaiser-Meyer-Olkin-Criteria (KMO) and performing Bartlett’s test of sphericity, principal component analyses (PCA) were used to extract factor loadings. Furthermore, confirmatory factor analyses (CFA) were used to test the known one-factor model of both instruments [30, 31]. Standardized factor loadings were extracted for each item and are displayed as supplemental resources (S2 Appendix).

Items were tested whether they had a suitable explanatory power using item-total correlation thresholds of <0.3 [32] and factor loadings <0.32 [33]. Further, items were checked for inter-item collinearity using a threshold for correlation coefficient > 0.7.

Discriminant validity was assessed to compare the sum scores of participants with and without known psychiatric conditions using the Mann-Whitney-*U* test. Furthermore, Spearman’s rho was used to calculate correlation coefficients between both psychometric scales and respective visual analog scales for depression, anxiety, and health-related quality of life (EQ-5D VAS score).

All descriptive and bivariate statistics were carried out using SPSS V29 (IBM Corp., Armonk NY) and the CFA was conducted using AMOS V29 (IBM Corp., Armonk NY).

### 2.6. Research ethics

The study received approval from the Corewell Health Institutional Review Board (Approval #: 2022–075). Only anonymized data were collected. Participants received written information and provided written consent prior to enrollment.

## 3. Results

### 3.1. Sample characteristics

Out of n = 133 recorded responses, 14 were excluded due to missing values resulting in a sample of n = 119. Participants were predominantly female (68.9%) and had a mean age of 40 years (SD 15.5). Almost half of the participants were recruited in the participating three family medicine clinics (49.6%, n = 59), and the remaining n = 60 participants (50.4%) were enrolled in other primary care clinics of the health care system through Kinyarwanda interpreter.

Only 10 (8.9%) participants reported having a known psychiatric condition, of which anxiety disorder (n = 6), PTSD, and Depression (each n = 4) were the most common. Approximately 17.8% of the participants reported having no school education and 42.9% reported speaking English “not at all”. Further sociodemographic information is outlined in Table 1.

**Table 1:** Participant characteristics.

Characteristic*		Female	Male	Total
		N = 82	N = 37	N = 119
		n (%)	n (%)	n (%)
Sociodemographic characteristics				
Age	$\bar{\text{x}}$ (SD)	38.6 (13.6)	43.3 (19.1)	40.0 (15.5)
	18–29 years	21 (26.3)	9 (26.5)	30 (26.3)
	30–49 years	45 (56.3)	13 (38.2)	58 (50.9)
	50+ years	14 (17.5)	12 (35.3)	26 (22.8)
Education	0–4 school years	27 (33.3)	5 (13.5)	32 (27.1)
	5–9 school years	20 (24.7)	12 (32.4)	32 (27.1)
	10+ school years	34 (42)	20 (54.1)	54 (45.8)
Time in the US	<1 year	12 (14.6)	10 (27)	22 (18.5)
	1–2 years	10 (12.2)	8 (21.6)	18 (15.1)
	2–3 years	5 (6.1)	1 (2.7)	6 (5)
	3–4 years	8 (9.8)	4 (10.8)	12 (10.1)
	4–5 years	19 (23.2)	4 (10.8)	23 (19.3)
	>5 years	28 (34.1)	10 (27)	38 (31.9)
English proficiency	not at all	38 (46.3)	13 (35.1)	51 (42.9)
	Beginner	27 (32.9)	11 (29.7)	38 (31.9)
	intermediate	13 (15.9)	12 (32.4)	25 (21)
	Advanced	1 (1.2)	0 (0)	1 (0.8)
	Fluent	3 (3.7)	1 (2.7)	4 (3.4)
Psychiatric condition		5 (6.6)	5 (13.9)	10 (8.9)
Psychometrics^#^				
EQ-5D VAS score	$\bar{\text{x}}$ (SD)	81.1 (23.8)	75 (20.6)	79.3 (22.9)
Self-Perceived Anxiety	VAS 0–100 $\bar{\text{x}}$ (SD)	4.3 (13.9)	15.7 (27.8)	10.5 (21.0)
Self-Perceived Depression	VAS 0–100 $\bar{\text{x}}$ (SD)	7.8 (18.6)	16.8 (24.8)	7.8 (19.8)
PHQ-9 Sum Score	0–27 $\bar{\text{x}}$ (SD)	1.7 (2.7)	2.9 (4.5)	2.0 (3.4)
GAD-7 Sum Score	0–21, $\bar{\text{x}}$ (SD)	1.0 (2.6)	1.4 (3.6)	1.1 (2.9)

* Missing age n = 5, Missing education n = 1, psychiatric condition missing n = 7, EQ-5D VAS missing n = 5, Anxiety VAS missing n = 2, Depression VAS missing = 1,

^#^ EQ-5D: Higher scores indicate better self-perceived health, all other scores: Higher scores indicate higher symptom burden.

### 3.2. Psychometrics and item characteristics

**Fig 1:**
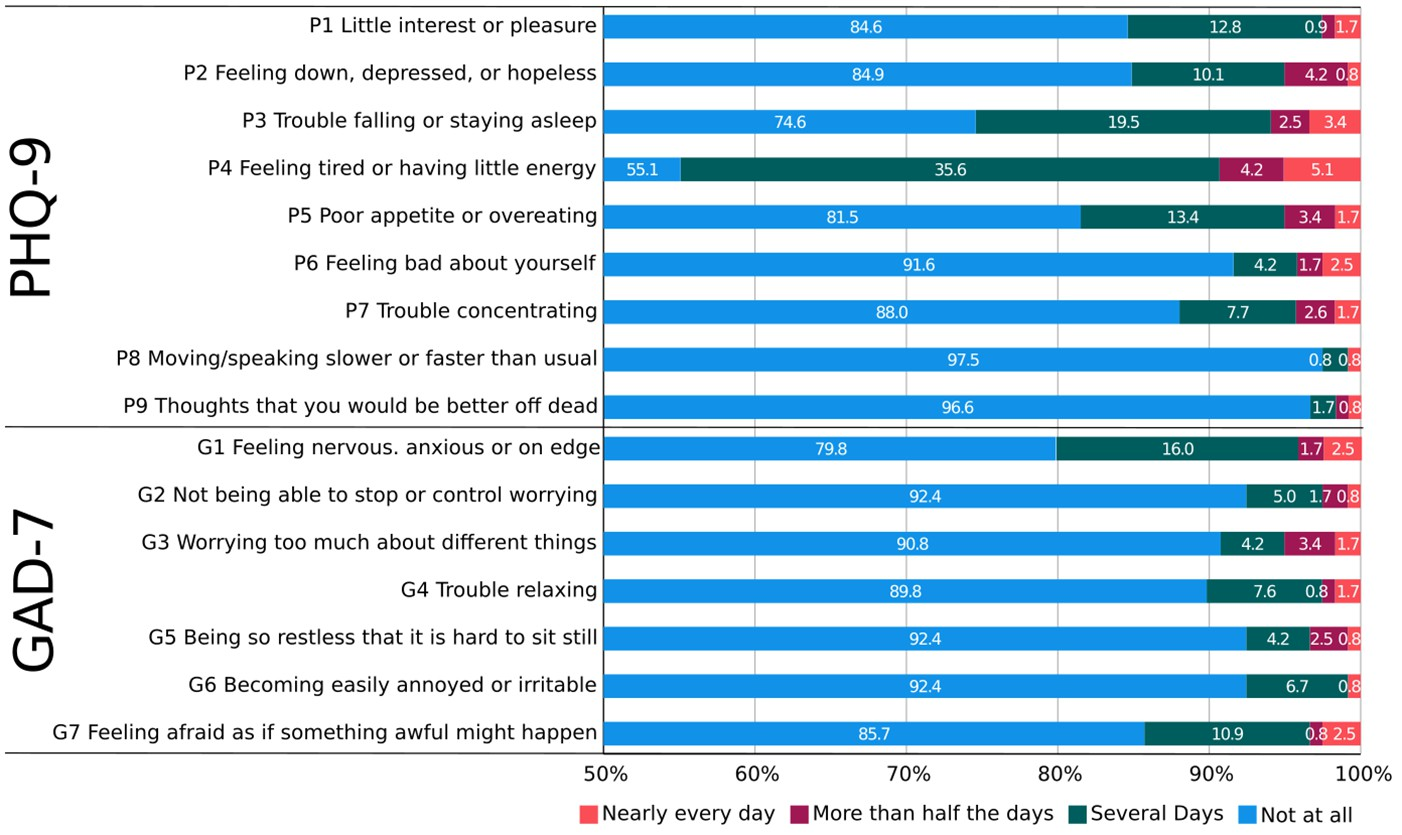
Participants’ responses.

Most participants displayed normal depression (PHQ-9 sum score 0–4, 89.9%) and anxiety levels (GAD-7 sum score 0–4, 95%). Sum scores of both parameters exhibited a left-skewed distribution with a skewness of 3.57 (standard error 0.22) and kurtosis of 18.02 (standard error 0.44) for the PHQ-9 and a skewness of 4.37 (standard error 0.22) and kurtosis of 21.07 (standard error 0.44) for the GAD-7. The distribution of responses is shown in Fig 1.

Inter-item correlation coefficients for the PHQ-9 and GAD-7 ranged between r = 0.06 to r = 0.65, and r = 0.23 to r = 0.65, respectively. A heatmap with correlation coefficients can be found as (S2 Appendix). PHQ-9 and GAD-7 sum scores were positively correlated (r = 0.59, p<0.001).

### 3.3. Reliability

PHQ-9 and GAD-7 displayed overall good internal consistency with a Cronbach’s α = 0.85 (McDonald’s Ω = 0.87) and α = 0.92 (McDonald’s Ω = 0.93), respectively. Item-total correlations ranged between 0.40 and 0.69 for the PHQ-9 and 0.68 and 0.85 for the GAD-7. Cronbach’s α did not significantly improve if items were removed from the scale.

In sensitivity analyses, lower α values for both PHQ-9 and GAD-7 were found for older respondents aged 50+ years, respondents who attempted 0–4 years in school, and respondents with lower self-perceived English proficiency and shorter stay in the US (Table 2).

**Table 2:** Internal consistency among subgroups.

Characteristic		PHQ-9	GAD-7
		α (95% CI)	α (95% CI)
Age group	18–29	0.79 (0.66–0.89)	0.94 (0.90–0.97)
	30–49	0.90 (0.86–0.94)	0.93 (0.90–0.95)
	50+	0.72 (0.52–0.86)	0.81 (0.68–0.91)
Education	0–4 school years	0.78 (0.64–0.88)	0.83 (0.72–0.91)
	5–9 school years	0.75 (0.60–0.87)	0.86 (0.77–0.92)
	10+ school years	0.91 (0.86–0.94)	0.94 (0.91–0.96)
Time in the US	0–3 years	0.66 (0.49–0.79)	0.56 (0.33–0.73)
	> 3 years	0.88 (0.83–0.92)	0.94 (0.92–0.96)
English proficiency	not at all	0.75 (0.62–0.84)	0.76 (0.64–0.85)
	beginner-fluent	0.91 (0.88–0.94)	0.95 (0.93–0.97)
Total		0.85 (0.81–0.89)	0.92 (0.89–0.94)

The Kaiser-Meyer-Olkin (KMO) value was 0.760 for PHQ-9 and Bartlett’s test of sphericity was significant (*χ*^2^ (36) = 552.53, p<0.001). Similar results were shown for the GAD-7 (KMO value: 0.873, Bartlett’s *χ*^2^ (21) = 612.56, p<0.001) suggesting both samples as appropriate for factor analyses. A principal component analysis for a single-factor design revealed factor loadings between 0.44 and 0.80 for the PHQ-9 and 0.76 to 0.90 for the GAD-7 (Table 3).

**Table 3:** Item characteristics, item-total-correlations, and factor loadings for PHQ-9 (N = 114) and GAD-7 (N = 118).

Item		Score		Item-Total correlation	α if item deleted	Factor Loading PCA
		Mean	SD			
P1	Little interest or pleasure in doing things.	0.19	0.53	0.60	0.83	0.70
P2	Feeling down, depressed, or hopeless.	0.22	0.56	0.69	0.82	0.79
P3	Trouble falling or staying asleep.	0.34	0.68	0.59	0.84	0.66
P4	Feeling tired or having little energy.	0.58	0.80	0.55	0.85	0.64
P5	Poor appetite or overeating.	0.25	0.59	0.40	0.85	0.44
P6	Feeling bad about yourself.	0.16	0.57	0.69	0.82	0.80
P7	Trouble concentrating.	0.18	0.56	0.64	0.83	0.74
P8	Moving/speaking slower or faster than usual.	0.04	0.31	0.56	0.84	0.72
P9	Thoughts that you would be better off dead.	0.06	0.36	0.66	0.84	0.80
G1	Feeling nervous, anxious or on edge.	0.27	0.62	0.68	0.92	0.76
G2	Not being able to stop or control worrying.	0.11	0.43	0.80	0.90	0.85
G3	Worrying too much about different things.	0.16	0.55	0.76	0.91	0.83
G4	Trouble relaxing.	0.14	0.49	0.73	0.91	0.81
G5	Being so restless that it is hard to sit still.	0.12	0.46	0.85	0.90	0.90
G6	Becoming easily annoyed or irritable.	0.09	0.37	0.77	0.91	0.84
G7	Feeling something awful might happen.	0.20	0.58	0.77	0.90	0.84

Using CFA, factor loadings ranged between 0.31 and 0.82 for the PHQ-9 and 0.73 and 0.89 for the GAD-7, however, model fit indices for CFAs were poor (S2 Appendix).

### 3.4. Discriminant validity

**Fig 2:**
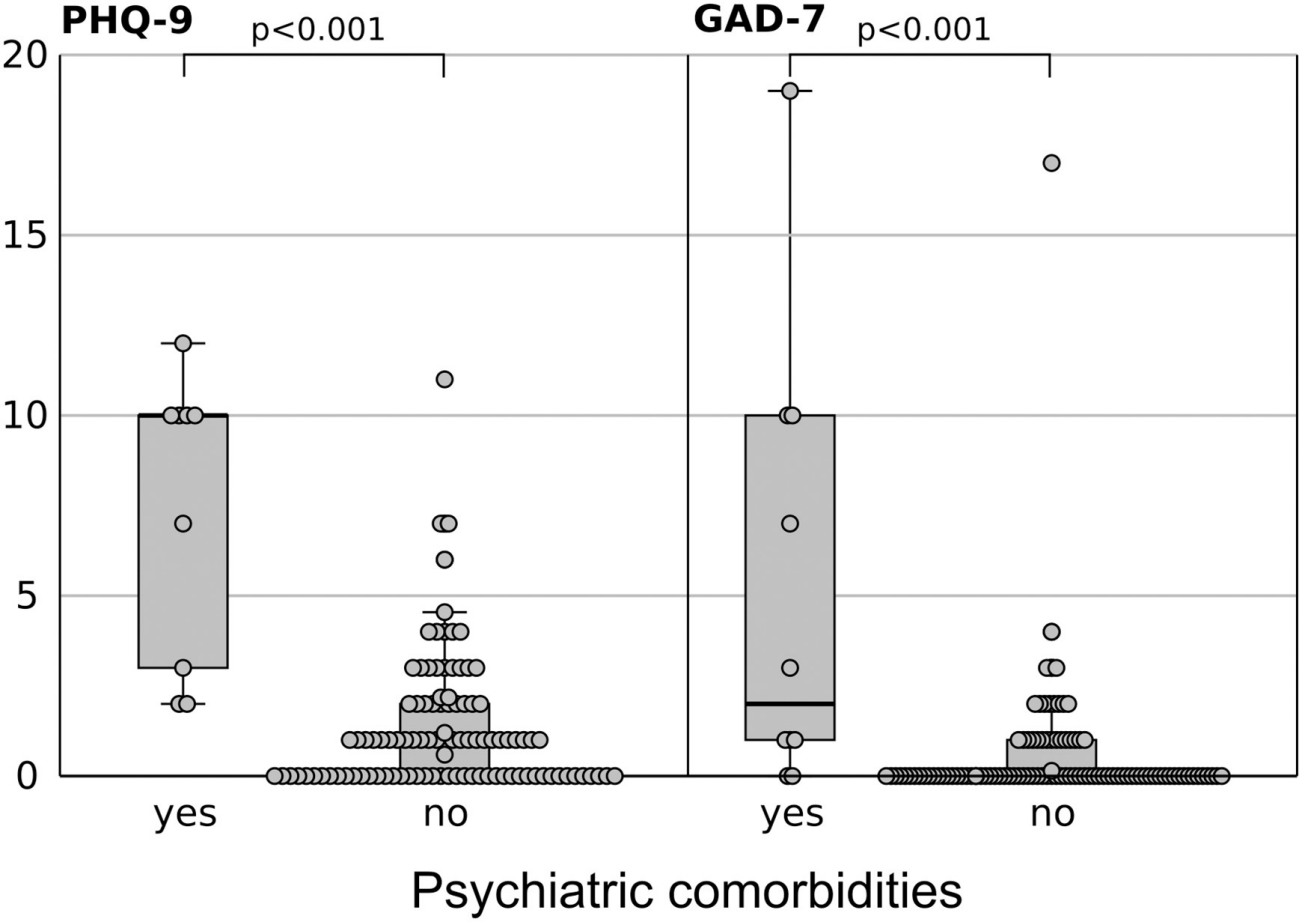
PHQ-9 and GAD-7 values among participants with preexisting psychiatric conditions (Mann-Whitney*-U* test).

Participants with psychiatric comorbidities had significantly higher scores on the PHQ-9 (mean 9.1 [SD 6.7]) compared to those without comorbidity (mean 1.3 [SD 1.8], p<0.001). The same pattern was observed for the GAD-7 scores, with those with comorbidities scoring higher (mean 5.2 [SD 6.3]) than those without (mean 0.6 [SD 1.8], p<0.001) (see Fig 2).

Self-perceived depression, as measured on a Visual Analog Scale (VAS), exhibited a strong positive correlation with the PHQ-9 sum score (r = 0.53, p<0.001). Similarly, self-perceived anxiety VAS scores showed a strong positive correlation with the GAD-7 sum score (r = 0.65, p<0.001).

Health-related quality of life using the EQ-5D VAS score showed a strong negative correlation with the PHQ-9 sum score (r = -0.59, p<0.001) and a moderate negative correlation with the GAD-7 sum score (r = -0.42, p<0.001).

### 3.5. Comments

Only few participants made written comments or annotations to the survey, mainly elaborating on why participants faced trouble falling asleep (P3), referring to environmental noise due to housing situation or busy roads.

## 4. Discussion

Studies validating psychometric instruments in a primary care situation for migrants and refugees are limited, especially for smaller language communities. The lack of availability of these instruments, however, is a key factor in insufficient mental health screening among refugee and migrant populations that are particularly affected by the trauma of relocation and mental health problems [34]. Research has demonstrated the practical clinical value of culturally adapted psychometric screening tools in accurately identifying and diagnosing mental health problems among refugee and migrant populations [35].

This work aimed to translate and culturally adapt the commonly used mental health screening instruments PHQ-9 and GAD-7 into the Kinyarwanda language and conduct a validation study by determining internal consistency, factorial structure, and discriminant validity in a cross-sectional sample of migrant and refugee patients seeking care in primary care clinics in the Midwest.

Our study underscores the importance of cultural adaptation beyond mere translation [18, 36]. Our approach to include a diverse committee of translators, clinicians, and researchers familiar with both knowledge in Rwandan culture as well as Western mental health concepts, of which all had hands-on experience in providing health care to Kinyarwanda-speaking communities was crucial to discussing cultural sensitivity. For example, our interpreters highlighted the stigma associated with mental health conditions in Kinyarwanda communities, or the absence of certain disease concepts (such as schizophrenia) leading to careful consideration of our word choice. Similar to other authors, we found that even basic terms like "mental health" could imply "madness" in direct translation, and pictorial metaphors like "feeling on edge" posed particular challenges [37]. These insights led to adaptations in the phrasing and presentation of items and were crucial in ensuring the PHQ-9 and GAD-7 were not just linguistically accurate, but culturally meaningful and relevant to our target population. Cognitive interviews with bilingual participants provided further insights into how mental health concepts were understood in the Kinyarwanda-speaking community [38].

We found for both instruments an overall good consistency; however, lower consistency levels were observed among patients who were new to the US, had lower English proficiency levels, or were older (50+ years). Particularly the PHQ-9 items on poor appetite/overeating and sleeping problems showed lower factor loadings. However, five items had factor loadings >0.6, suggesting the overall measured factor as reliable [39].

Some participants made annotations to the survey, indicating that their impaired sleep conditions were due to environmental noise exposure that was associated with their housing situation. Research has indicated that newly arrived refugees and migrants are often accommodated in shelters, boarding houses, or substandard apartment buildings that lack privacy and are often exposed to noise that may impair their sleep [40, 41]. Similarly, newly arriving refugees often face considerable challenges in retaining their food-related practices [42, 43], with particular hardships in acquiring goods commonly consumed in their country of origin while facing a limited budget [44]. As a result newcomers to the US often face food insecurity [45], which is often further aggravated by language barriers [46]. These aspects likely contribute to a feeling of decreased appetite, especially among newly arrived migrants without being a clear sign that would indicate an impairment of mental health. This may help explain the lower consistency among newcomers and lower factor loadings of the respective items in this cohort.

The rather small number of incomplete surveys suggests that the survey questions were understood well by various patient groups, including those with limited education. The quality of the translation was confirmed in cognitive interviews.

While we have performed a confirmatory factor analysis, the indicated model of fit indices were poor. It is likely that this is due to a combination of lower factor loadings among some of the previously discussed items and the vulnerability of goodness of fit indices when analyzing smaller samples [47].

Although we were not able to validate our instruments against an established gold standard, we found that respective sum scores correlated well with visual analog scales for health-related quality of life, depression, and anxiety. Furthermore, patients with a known history of mental health problems scored significantly higher for both instruments.

This study comes with further limitations that need to be considered when interpreting the results. Although our sample size met the suggested item-to-response ratios, a considerably larger sample size would have led to more robust findings, especially among subgroups of the sample, and might have yielded better goodness-of-fit indices in the confirmatory factor analyses. The majority of enrolled patients in our sample indicated no or few mental health problems thus reducing variability within our sample. This could be addressed with either a larger sample or a sample that includes more people with such problems, e.g. recruited through mental health providers.

Recently other psychometric instruments in the Kinyarwanda language have been introduced, such as the Beck Depression Inventory [48] or the Mini International Neuropsychiatric Interview [49], however, these were not used in our study as they have not been validated in the respective setting of primary care clinics in the US.

## 5. Conclusion

This study provides the first validated Kinyarwanda versions of the PHQ-9 and GAD-7 for use in US primary care settings, offering essential tools for mental health screening in Kinyarwanda-speaking communities. While these instruments demonstrate good internal consistency and discriminant validity, further research is needed. Future studies should investigate validity against standard diagnostic interviews in larger, more diverse samples, including patients from mental health clinics and psychiatric hospitals. Such research could establish diagnostic accuracy and determine population-specific cut-off scores. Despite these limitations, our culturally adapted PHQ-9 and GAD-7 represent significant steps towards more equitable and culturally competent mental health care for Kinyarwanda speakers in primary care settings.

## Supporting information

S1 Appendix PHQ-9 and GAD-7 in Kinyarwanda. (DOCX)

S2 Appendix Heatmap/CFA. (DOCX)

S3 Appendix STROBE checklist. (DOCX)

S1 Dataset PHQ-9. (XLSX)

S2 Dataset GAD-7. (XLSX)

## References

[1] Depression and Other Common Mental Disorders: Global Health Estimates. World Health Organization; 2017. https://iris.who.int/bitstream/handle/10665/254610/WHO-MSD-MER-2017.2-eng.pdf

[2] Weinberger AH, Gbedemah M, Martinez AM, Nash D, Galea S, Goodwin RD. Trends in depression prevalence in the USA from 2005 to 2015: widening disparities in vulnerable groups. Psychol Med. 2018;48: 1308–1315. doi: 10.1017/S0033291717002781 29021005

[3] Ösby U, Brandt L, Correia N, Ekbom A, Sparén P. Excess Mortality in Bipolar and Unipolar Disorder in Sweden. Arch Gen Psychiatry. 2001;58: 844. doi: 10.1001/archpsyc.58.9.844 11545667

[4] Lépine J-P, Briley M. The increasing burden of depression. NDT. 2011; 3. doi: 10.2147/NDT.S19617 21750622 PMC3131101

[5] Barth J, Schumacher M, Herrmann-Lingen C. Depression as a Risk Factor for Mortality in Patients With Coronary Heart Disease: A Meta-analysis. Psychosomatic Medicine. 2004;66: 802–813. doi: 10.1097/01.psy.0000146332.53619.b2 15564343

[6] Birk JL, Kronish IM, Moise N, Falzon L, Yoon S, Davidson KW. Depression and multimorbidity: Considering temporal characteristics of the associations between depression and multiple chronic diseases. Health Psychology. 2019;38: 802–811. doi: 10.1037/hea0000737 31008648 PMC6706317

[7] Egede LE, Zheng D, Simpson K. Comorbid Depression is Associated With Increased Health Care Use and Expenditures in Individuals With Diabetes. Diabetes Care. 2002;25: 464–470. doi: 10.2337/diacare.25.3.464 11874931

[8] Siu AL, and the US Preventive Services Task Force (USPSTF), Bibbins-Domingo K, Grossman DC, Baumann LC, Davidson KW, et al. Screening for Depression in Adults: US Preventive Services Task Force Recommendation Statement. JAMA. 2016;315: 380. doi: 10.1001/jama.2015.18392 26813211

[9] O’Connor E, Henninger M, Perdue LA, Coppola EL, Thomas R, Gaynes BN. Screening for Depression, Anxiety, and Suicide Risk in Adults: A Systematic Evidence Review for the U.S. Preventive Services Task Force. Agency for Healthcare Research and Quality; 2022. Report No.: AHRQ Publication No. 22-05295-EF-1. 37406149

[10] Tonui BC. The cultural perceptions on mental health and post-resettlement challenges among Rwandan refugees in the U.S. Psychiatry Research. 2022;313: 114642. doi: 10.1016/j.psychres.2022.114642 35609498

[11] Mesa-Vieira C, Haas AD, Buitrago-Garcia D, Roa-Diaz ZM, Minder B, Gamba M, et al. Mental health of migrants with pre-migration exposure to armed conflict: a systematic review and meta-analysis. The Lancet Public Health. 2022;7: e469–e481. doi: 10.1016/S2468-2667(22)00061-5 35487232

[12] Abubakar I, Aldridge RW, Devakumar D, Orcutt M, Burns R, Barreto ML, et al. The UCL–Lancet Commission on Migration and Health: the health of a world on the move. The Lancet. 2018;392: 2606–2654. doi: 10.1016/S0140-6736(18)32114-7 30528486 PMC7612863

[13] Müller F, Kleinert E, Hillermann N, Simmenroth A, Hummers E, Scharff AZ, et al. Disease burden in a large cohort of asylum seekers and refugees in Germany. J Glob Health. 2021;11: 04002. doi: 10.7189/jogh.11.04002 33643633 PMC7897448

[14] Kirmayer LJ. Cultural variations in the clinical presentation of depression and anxiety: implications for diagnosis and treatment. J Clin Psychiatry. 2001;62 Suppl 13: 22–28; discussion 29–30. 11434415

[15] Kleinert E, Müller F, Furaijat G, Hillermann N, Jablonka A, Happle C, et al. Does refugee status matter? Medical needs of newly arrived asylum seekers and resettlement refugees—a retrospective observational study of diagnoses in a primary care setting. Confl Health. 2019;13: 39. doi: 10.1186/s13031-019-0223-z 31452677 PMC6700982

[16] Rahal S. Michigan becomes haven for Congo refugees. In: The Detroit News [Internet]. 12 Apr 2019 [cited 25 Mar 2024]. https://eu.detroitnews.com/story/news/local/michigan/2019/04/12/michigan-becomes-haven-congo-refugees/3287895002/

[17] Byrow Y, Pajak R, Specker P, Nickerson A. Perceptions of mental health and perceived barriers to mental health help-seeking amongst refugees: A systematic review. Clinical Psychology Review. 2020;75: 101812. doi: 10.1016/j.cpr.2019.101812 31901882

[18] Bhui K, Mohamud S, Warfa N, Craig TJ, Stansfeld SA. Cultural adaptation of mental health measures: Improving the quality of clinical practice and research. Br J Psychiatry. 2003;183: 184–186. doi: 10.1192/bjp.183.3.184 12948986

[19] Kroenke K, Spitzer RL, Williams JBW. The PHQ-9: Validity of a brief depression severity measure. J Gen Intern Med. 2001;16: 606–613. doi: 10.1046/j.1525-1497.2001.016009606.x 11556941 PMC1495268

[20] Sebera F, Vissoci JRN, Umwiringirwa J, Teuwen DE, Boon PE, Dedeken P. Validity, reliability and cut-offs of the Patient Health Questionnaire-9 as a screening tool for depression among patients living with epilepsy in Rwanda. Tran TD, editor. PLoS ONE. 2020;15: e0234095. doi: 10.1371/journal.pone.0234095 32530968 PMC7292570

[21] Spitzer RL, Kroenke K, Williams JBW, Löwe B. A Brief Measure for Assessing Generalized Anxiety Disorder: The GAD-7. Arch Intern Med. 2006;166: 1092. doi: 10.1001/archinte.166.10.1092 16717171

[22] von Elm E, Altman DG, Egger M, Pocock SJ, Gøtzsche PC, Vandenbroucke JP, et al. The Strengthening the Reporting of Observational Studies in Epidemiology (STROBE) statement: guidelines for reporting observational studies. J Clin Epidemiol. 2008;61: 344–349. doi: 10.1016/j.jclinepi.2007.11.008 18313558

[23] Valdez D, Montenegro MS, Crawford BL, Turner RC, Lo W-J, Jozkowski KN. Translation frameworks and questionnaire design approaches as a component of health research and practice: A discussion and taxonomy of popular translation frameworks and questionnaire design approaches. Social Science & Medicine. 2021;278: 113931. doi: 10.1016/j.socscimed.2021.113931 33905986

[24] Behr D. Translating questionnaires for cross-national surveys: A description of a genre and its particularities based on the ISO 17100 categorization of translator competences. Trans-Int. 2018;10: 5–20. doi: 10.12807/ti.110202.2018.a02

[25] Schwarz N. Cognitive aspects of survey methodology. Appl Cognit Psychol. 2007;21: 277–287. doi: 10.1002/acp.1340

[26] Tourangeau R, Rips LJ, Rasinski K. The Psychology of Survey Response. 1st ed. Cambridge University Press; 2000.

[27] Rabin R, Charro FD. EQ-SD: a measure of health status from the EuroQol Group. Annals of Medicine. 2001;33: 337–343. doi: 10.3109/07853890109002087 11491192

[28] Huang Z, Kohler IV, Kämpfen F. A Single-Item Visual Analogue Scale (VAS) Measure for Assessing Depression Among College Students. Community Ment Health J. 2020;56: 355–367. doi: 10.1007/s10597-019-00469-7 31531784

[29] MacCallum RC, Widaman KF, Zhang S, Hong S. Sample size in factor analysis. Psychological Methods. 1999;4: 84–99. doi: 10.1037/1082-989X.4.1.84

[30] Gelaye B, Williams MA, Lemma S, Deyessa N, Bahretibeb Y, Shibre T, et al. Validity of the Patient Health Questionnaire-9 for depression screening and diagnosis in East Africa. Psychiatry research. 2013;210: 653–661. doi: 10.1016/j.psychres.2013.07.015 23972787 PMC3818385

[31] Johnson SU, Ulvenes PG, Øktedalen T, Hoffart A. Psychometric Properties of the General Anxiety Disorder 7-Item (GAD-7) Scale in a Heterogeneous Psychiatric Sample. Frontiers in psychology. 2019;10: 1713. doi: 10.3389/fpsyg.2019.01713 31447721 PMC6691128

[32] Cristobal E, Flavián C, Guinalíu M. Perceived e–service quality (PeSQ). Managing Service Quality: An International Journal. 2007;17: 317–340. doi: 10.1108/09604520710744326

[33] Comrey AL, Lee HB. A first course in factor analysis. Second edition. New York: Psychology Press; 2016.

[34] Müller F, Abdelnour AM, Rutaremara DN, Arnetz JE, Achtyes ED, Alshaarawy O, et al. Mental Health Screening Differences in Non-English Speaking Patients: Results From a Retrospective Cohort Study. J Prim Care Community Health. 2023;14: 21501319231200304. doi: 10.1177/21501319231200304 37714820 PMC10504842

[35] Savin D, Seymour DJ, Littleford LN, Bettridge J, Giese A. Findings from Mental Health Screening of Newly Arrived Refugees in Colorado. Public Health Rep. 2005;120: 224–229. doi: 10.1177/003335490512000303 16134561 PMC1497730

[36] Beaton DE, Bombardier C, Guillemin F, Ferraz MB. Guidelines for the Process of Cross-Cultural Adaptation of Self-Report Measures. Spine. 2000;25. https://journals.lww.com/spinejournal/fulltext/2000/12150/guidelines_for_the_process_of_cross_cultural.14.aspx 10.1097/00007632-200012150-00014 11124735

[37] Kohrt BA, Luitel NP, Acharya P, Jordans MJD. Detection of depression in low resource settings: validation of the Patient Health Questionnaire (PHQ-9) and cultural concepts of distress in Nepal. BMC Psychiatry. 2016;16: 58. doi: 10.1186/s12888-016-0768-y 26951403 PMC4782581

[38] Willis GB, Miller K. Cross-Cultural Cognitive Interviewing: Seeking Comparability and Enhancing Understanding. Willis GB, Miller K, editors. Field Methods. 2011;23: 331–341. doi: 10.1177/1525822X11416092

[39] Field A. Discovering statistics using SPSS for Windows: Advanced techniques for the beginner. Repr. London: Sage Publs; 2004.

[40] Bakker L, Cheung SY, Phillimore J. The Asylum–Integration Paradox: Comparing Asylum Support Systems and Refugee Integration in The Netherlands and the UK. International Migration. 2016;54: 118–132. doi: 10.1111/imig.12251

[41] Gewalt SC, Berger S, Ziegler S, Szecsenyi J, Bozorgmehr K. Psychosocial health of asylum seeking women living in state-provided accommodation in Germany during pregnancy and early motherhood: A case study exploring the role of social determinants of health. PloS one. 2018;13: e0208007. doi: 10.1371/journal.pone.0208007 30592728 PMC6310271

[42] Hersey J, Anliker J, Miller C, Mullis RM, Daugherty S, Das S, et al. Food shopping practices are associated with dietary quality in low-income households. Journal of nutrition education. 2001;33 Suppl 1: S16–26. doi: 10.1016/s1499-4046(06)60066-3 12857541

[43] Sellen D, Tedstone A, Kerhsen J. Food in the Migrant Experience. 2002.

[44] Drewnowski A, Specter SE. Poverty and obesity: the role of energy density and energy costs. The American journal of clinical nutrition. 2004;79: 6–16. doi: 10.1093/ajcn/79.1.6 14684391

[45] Hadley C, Sellen D. Food security and child hunger among recently resettled Liberian refugees and asylum seekers: a pilot study. Journal of immigrant and minority health. 2006;8: 369–375. doi: 10.1007/s10903-006-9007-9 16924410

[46] Himmelgreen D, Pérez-Escamilla R, Segura-Millán S, Peng Y-K, Gonzalez A, Singer M, et al. Food Insecurity Among Low-Income Hispanics in Hartford, Connecticut: Implications for Public Health Policy. Human Organization. 2000;59: 334–342. doi: 10.17730/humo.59.3.76557m317748l414

[47] Sun J. Assessing Goodness of Fit in Confirmatory Factor Analysis. Measurement and Evaluation in Counseling and Development. 2005;37: 240–256. doi: 10.1080/07481756.2005.11909764

[48] Dedeken P, Nickenig Vissoci JR, Sebera F, Boon PAJM, Rutembesa E, Teuwen DE. Validity, Reliability, and Diagnostic Cut-off of the Kinyarwandan Version of the Hamilton Depression Rating Scale in Rwanda. Frontiers in psychology. 2020;11: 1343. doi: 10.3389/fpsyg.2020.01343 32719632 PMC7350898

[49] Kayiteshonga Y, Sezibera V, Mugabo L, Iyamuremye JD. Prevalence of mental disorders, associated co-morbidities, health care knowledge and service utilization in Rwanda—towards a blueprint for promoting mental health care services in low- and middle-income countries? BMC public health. 2022;22: 1858. doi: 10.1186/s12889-022-14165-x 36199102 PMC9533613

