# Supplementary material for: Translation, cultural adaptation, and validation of the PHQ-9 and GAD-7 in Kinyarwanda for primary care in the United States
Source: PLoS One. 2024 Oct 17;19(10):e0302953. doi: 10.1371/journal.pone.0302953 (PMC11486410; doi:10.1371/journal.pone.0302953)
Supplement: S1 Appendix — (DOCX) [file pone.0302953.s001.docx]

# S1 Appendix: PHQ-9 / GAD-7 in Kinyarwanda

| **Mubyumweru 2 bishize, ni inshuro zingahe waba warabangamiwe na bimwe mubibazo bikurikira?** *Koresha kano kamenyetso ⌧ werekana igisubizo cyawe.* | Nta nagato | Iminsi mike | Birenze iminsi myinshi iruta kimwe cya kabiri cy’ibyumweru bibiri bishize | Hafi ya buri munsi |
| --- | --- | --- | --- | --- |
|  | 0 | 1 | 2 | 3 |
| **P1.** Kudashishikarira ibyo ukora cyangwa ntushimishwe nabyo.  (Little interest or pleasure in doing things) | 🞏 | 🞏 | 🞏 | 🞏 |
| **P2.** Kumva utameze neza, kugira agahinda gakabije cyangwa se kwiheba.  (Feeling down, depressed, or hopeless) | 🞏 | 🞏 | 🞏 | 🞏 |
| **P3.** Kubura ibitotsi cyangwa se kudashobora gusinzira, cyangwa se gusinzira bikabije.  (Trouble falling or staying asleep, or sleeping too much) | 🞏 | 🞏 | 🞏 | 🞏 |
| **P4.** Kumva unaniwe cyangwa ufite imbaraga nkeya cyane.  (Feeling tired or having little energy) | 🞏 | 🞏 | 🞏 | 🞏 |
| **P5.** Kumva udashaka kurya cyangwa kurya birenze urugero.  (Poor appetite or overeating) | 🞏 | 🞏 | 🞏 | 🞏 |
| **P6.** Kwitekerezaho cyane kandi nabi, kumva nta kamaro ufite, kumva ntacyo wimariye cyangwa umariye umuryango wawe. (Feeling bad about yourself — or that you are a failure or have let yourself or your family down) | 🞏 | 🞏 | 🞏 | 🞏 |
| **P7.** Kunanirwa gukurikirina ibintu, harimo nko kunanirwa gusoma ibinyamakuru byanditse cyangwa se kureba inyerekamashusho (Tereviziyo). (Trouble concentrating on things, such as reading the newspaper or watching television) | 🞏 | 🞏 | 🞏 | 🞏 |
| **P8.** Kugenda cyangwa ukavuga buhoro kuburyo budasanzwe bikagaragarira abandi cyangwa se bitandukanye ukaba wihuta cyane, kubura umutuzo, ugahorana umuvuduko ukabije (udasanzwe) mu byo ukora. (Moving or speaking so slowly that other people could have noticed? Or the opposite — being so fidgety or restless that you have been moving around a lot more than usual) | 🞏 | 🞏 | 🞏 | 🞏 |
| **P9.** Gutekereza ko wakumva umeze neza wapfuye cyangwa se ukumva ushaka kwigirira nabi. (Thoughts that you would be better off dead or of hurting yourself in some way) | 🞏 | 🞏 | 🞏 | 🞏 |
| **G1.** Guhorana impungenge, guhangayika cyangwa se kudashyira umutima hamwe. (Feeling nervous, anxious or on edge) | 🞏 | 🞏 | 🞏 | 🞏 |
| **G2.** Kudashobora guhagarika ibitekerezo byo kugira ubwoba. (Not being able to stop or control worrying) | 🞏 | 🞏 | 🞏 | 🞏 |
| **G3.** Kugira ubwoba bwinshi cyane kubintu bitandukanye. (Worrying too much about different things) | 🞏 | 🞏 | 🞏 | 🞏 |
| **G4.** Kutabasha kuruhuka. (Trouble relaxing) | 🞏 | 🞏 | 🞏 | 🞏 |
| **G5.** Kumva utabasha kuruhuka ukananirwa kwicara hamwe ngo utuze. (Being so restless that it is hard to sit still) | 🞏 | 🞏 | 🞏 | 🞏 |
| **G6.** Kurakazwa n‘ubusa, kumva ubangamiwe n‘ibintu bidafite agaciro. (Becoming easily annoyed or irritable) | 🞏 | 🞏 | 🞏 | 🞏 |
| **G7.** Kugira ubwoba ko hari ikintu kibi gishobora kuba.. (Feeling afraid as if something awful might happen) | 🞏 | 🞏 | 🞏 | 🞏 |

**Niba hari icyo watoranyije muri bino bibazo, ese bigukomereye kuruhe rugero aho ibi bibazo bituma udashobora gukora akazi kawe, gutunganya imirimo yo murugo cyangwa se n'imibanire yawe n’abandi?** (If you checked off any problems, how difficult have these problems made it for you to do your work, take care of things at home, or get along with other people?)

| Ntago bikomeye | Bikomeyeho gato | Birakomeye | Birakomeye cyane |
| --- | --- | --- | --- |
| 🞏 | 🞏 | 🞏 | 🞏 |
| Not difficult at all | Somewhat difficult | Very difficult | Extremely difficult |

Usabwe gusubiza ururupapuro wujuje kubakozi b’ibitaro.

Murakoze cyane!!
