# Supplementary material for: Translation, cultural adaptation, and validation of the PHQ-9 and GAD-7 in Kinyarwanda for primary care in the United States
Source: PLoS One. 2024 Oct 17;19(10):e0302953. doi: 10.1371/journal.pone.0302953 (PMC11486410; doi:10.1371/journal.pone.0302953)
Supplement: S2 Appendix — (DOCX) [file pone.0302953.s002.docx]

# S2 Appendix: Heatmap/CFA

|  | P1 | P2 | P3 | P4 | P5 | P6 | P7 | P8 | P9 | G1 | G2 | G3 | G4 | G5 | G6 | G7 |
| --- | --- | --- | --- | --- | --- | --- | --- | --- | --- | --- | --- | --- | --- | --- | --- | --- |
| P1 |  | .49^**^ | .24^*^ | .28^**^ | .20^*^ | .38^**^ | .32^**^ | .25^**^ | .20^*^ | .29^**^ | .24^**^ | .43^**^ | .35^**^ | .44^**^ | .33^**^ | .51^**^ |
| P2 | .49^**^ |  | .37^**^ | .35^**^ | .17 | .65^**^ | .45^**^ | .25^**^ | .34^**^ | .30^**^ | .36^**^ | .47^**^ | .42^**^ | .44^**^ | .61^**^ | .52^**^ |
| P3 | .26^*^ | .37^**^ |  | .34^**^ | .26^**^ | .28^**^ | .41^**^ | .06 | .13 | .33^**^ | .23^*^ | .17 | .36^**^ | .38^**^ | .37^**^ | .12 |
| P4 | .28^**^ | .35^**^ | .34^**^ |  | .37^**^ | .32^**^ | .34^**^ | .10 | .30^**^ | .37^**^ | .27^**^ | .17 | .38^**^ | .37^**^ | .29^**^ | .31^**^ |
| P5 | .20^*^ | .17 | .26^**^ | .37^**^ |  | .32^**^ | .45^**^ | .19^*^ | .14 | .39^**^ | .27^**^ | .30^**^ | .29^**^ | .37^**^ | .19^*^ | .07 |
| P6 | .38^**^ | .65^**^ | .28^**^ | .32^**^ | .32^**^ |  | .55^**^ | .34^**^ | .64^**^ | .42^**^ | .50^**^ | .56^**^ | .31^**^ | .50^**^ | .50^**^ | .42^**^ |
| P7 | .32^**^ | .45^**^ | .41^**^ | .34^**^ | .45^**^ | .55^**^ |  | .30^**^ | .39^**^ | .35^**^ | .31^**^ | .36^**^ | .25^**^ | .31^**^ | .34^**^ | .17 |
| P8 | .25^**^ | .25^**^ | .06 | .10 | .19^*^ | .34^**^ | .30^**^ |  | .57^**^ | .21^*^ | .36^**^ | .34^**^ | .31^**^ | .37^**^ | .37^**^ | .28^**^ |
| P9 | .20^*^ | .34^**^ | .13 | .30^**^ | .14 | .64^**^ | .39^**^ | .57^**^ |  | .29^**^ | .49^**^ | .45^**^ | .26^**^ | .48^**^ | .49^**^ | .35^**^ |
| G1 | .29^**^ | .30^**^ | .33^**^ | .37^**^ | .39^**^ | .42^**^ | .35^**^ | .21^*^ | .29^**^ |  | .45^**^ | .32^**^ | .23^*^ | .55^**^ | .38^**^ | .37^**^ |
| G2 | .24^**^ | .36^**^ | .23^*^ | .27^**^ | .27^**^ | .50^**^ | .31^**^ | .36^**^ | .49^**^ | .45^**^ |  | .59^**^ | .35^**^ | .54^**^ | .54^**^ | .46^**^ |
| G3 | .43^**^ | .47^**^ | .17 | .17 | .30^**^ | .56^**^ | .36^**^ | .34^**^ | .45^**^ | .33^**^ | .59^**^ |  | .31^**^ | .49^**^ | .59^**^ | .56^**^ |
| G4 | .35^**^ | .42^**^ | .36^**^ | .38^**^ | .29^**^ | .31^**^ | .25^**^ | .31^**^ | .26^**^ | .23^*^ | .35^**^ | .31^**^ |  | .47^**^ | .45^**^ | .44^**^ |
| G5 | .44^**^ | .44^**^ | .38^**^ | .37^**^ | .37^**^ | .50^**^ | .31^**^ | .37^**^ | .48^**^ | .55^**^ | .54^**^ | .49^**^ | .47^**^ |  | .65^**^ | .54^**^ |
| G6 | .33^**^ | .61^**^ | .37^**^ | .29^**^ | .19* | .50^**^ | .34^**^ | .37^**^ | .49^**^ | .38^**^ | .54^**^ | .59^**^ | .45^**^ | .65^**^ |  | .54^**^ |
| G7 | .51^**^ | .52^**^ | .12 | .31^**^ | .07 | .42^**^ | .17 | .28^**^ | .35^**^ | .37^**^ | .46^**^ | .56^**^ | .44^**^ | .54^**^ | .54^**^ |  |

Figure S1: Heatmap of correlation coefficients (Spearman’s rho) of psychometric items. ^*^ p<0.05, ^**^p<0.01

**
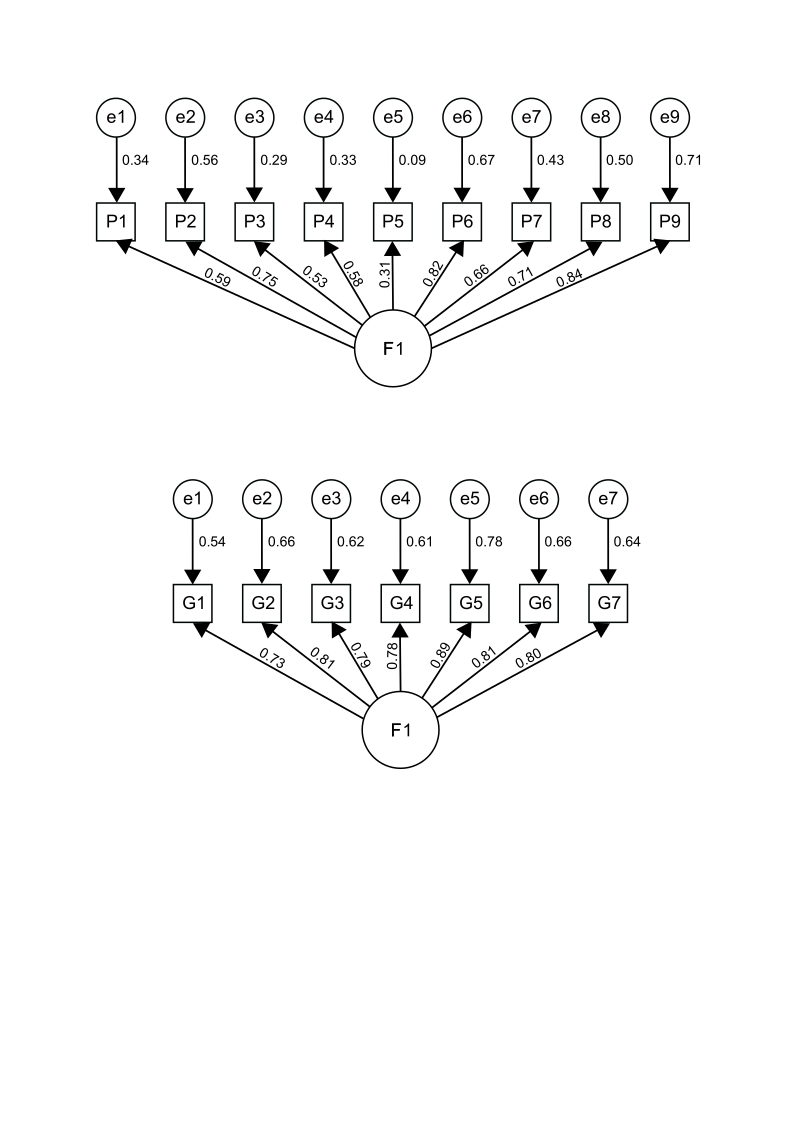
**

Figure S2: Model of Confirmatory Factor Analyses of PHQ-9 with standardized estimates (N=114).

**
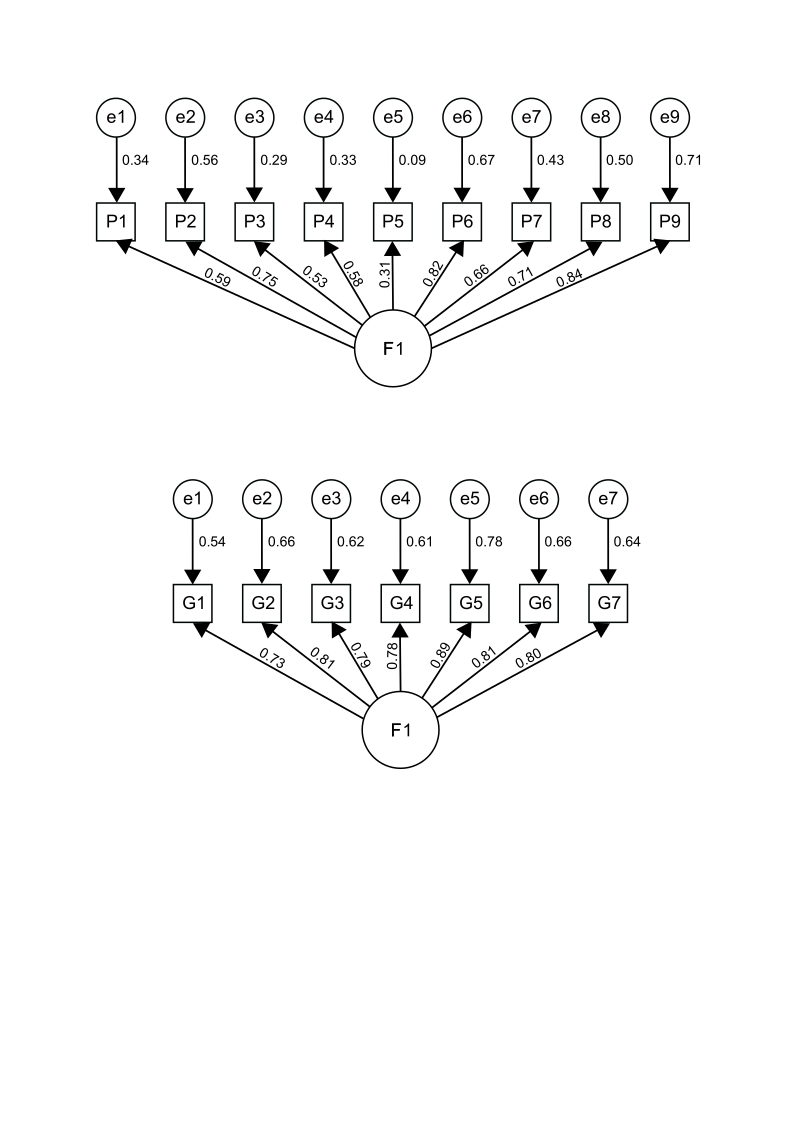
**

Figure S3: Model of Confirmatory Factor Analyses of GAD-7 with standardized estimates (N=118).

|  | PHQ-9 (N=114) | GAD-7 (N=118) |
| --- | --- | --- |
| CMIN/df | 7.02 | 5.51 |
| CFI | 0.70 | 0.90 |
| TLI | 0.60 | 0.84 |
| SRMR | 0.01 | 0.05 |
| RMSEA | 0.363 | 0.20 |

Table S1: CFA Model Fit Indices
