# Supplementary material for: Translation, cultural adaptation, and validation of the PHQ-9 and GAD-7 in Kinyarwanda for primary care in the United States
Source: PLoS One. 2024 Oct 17;19(10):e0302953. doi: 10.1371/journal.pone.0302953 (PMC11486410; doi:10.1371/journal.pone.0302953)
Supplement: Attachment — Submitted filename: Review feedback.docx [file pone.0302953.s006.docx]

**Review feedback**

I thank the authors for translating and validating mental health assessment tools for use in a minority group. The lack of appropriate tools has remained a big challenge to assessing and treating of depression in some contexts, thus, the study is relevant. However, the study can benefit from a few adjustments as suggested below.

Abstract. Line 33. Mention the number and category of experts who culturally translated the tools

Abstract Line 44: State the practical implications for the translation and validation

Introduction Line 67: Authors state that the PHQ-9 has been translated and evaluated among patients with epilepsy in Rwanda. My understanding is that the tool was used as a mental health screening tool among patients with epilepsy. If this was the case, then authors should provide a clear justification for a new translation and evaluation among the same population.

Line 70: The significance of validating tools is not well emphasized in the background. A strong case can be built

Methods

Line 80: Authors report using the STROBE guidelines however the checklist is not part of the supporting file

Lines 85-87, authors note ‘All commonly used translation and adaption frameworks entail rigorous steps of planning, parallel translations, review, adjudication, pilot-testing, revision, and documentation, that can be iterated as necessary’. This is a rigorous procedure in translation and cultural adaptions, but in the current study, apart from the translation process, other steps are not comprehensively reported. For example, who participated in the review? Why them? What did they review and what are the outcomes? The strength of adaptation is in the details. Authors need to give all the details of what was done and how it was done across all these processes.

159: The study setting is not explicitly explained. Additional details about the setting would help the reader understand better. More details about the Rwandese community in Michigan can help the reader relate better

Line 172: Authors treat exclusion criteria to be a total opposite of inclusion. Essentially exclusion means that the participant would qualify to participate but because of a certain issues or factor, they get excluded. Being 17 and below means that the participant was not qualified to participate from the onset, thus, that cannot be a criterion for exclusion

Line 178: Did consent happen just by reading the research objectives? Was it written or verbal consent? Secondly, in which language was ‘the paper-pencil survey’ which participants read and agreed to participate? Being a study conducted among a minority group, the consent procedure should be elaborate enough.

Line 203: Authors should report the exact sample size the used

Discussion

Overall, cultural adaptation is not sufficiently discussed. This is also a problem of conceptualization. Authors seem to have simplified translation to represent cultural adaptation. While the former is a process in adaptation, the latter is vast and need to be treated as a comprehensive process. Translation does not automatically represent adaptation although it can be part of the process.

Finally, authors need to include a list of supporting materials at the end of the manuscript
