# Supplementary material for: Translation, cultural adaptation, and validation of the PHQ-9 and GAD-7 in Kinyarwanda for primary care in the United States
Source: PLoS One. 2024 Oct 17;19(10):e0302953. doi: 10.1371/journal.pone.0302953 (PMC11486410; doi:10.1371/journal.pone.0302953)
Supplement: Attachment — Submitted filename: Comments to the Authors.docx [file pone.0302953.s007.docx]

Comments to the Authors

I reviewed this paper and found it interesting, particularly considering that depression and anxiety are major public health issues. As such, this manuscript deserves publication. However, the following issues need to be addressed:

Introduction

[1] The first paragraph needs to provide a brief overview of the epidemiology of depression and anxiety, highlighting their importance as public health issues.

[2] The second paragraph needs to justify why mental health issues disproportionately impact refugee populations.

Methods

[3] In the last paragraph of the ‘Translation and Cultural Adaptation’ section, it states that “the notes from the cognitive interviews and the results from the back-translation were then discussed in a final consensus meeting and the survey was finalized." You need to clarify whether both the forward and backwards translators were involved. If not, you need to clarify how you managed any inconsistencies between the forward and backwards translations.

[4] Sampling methods for how the participants were included in the study need to be addressed. Is there any randomisation? If so, what were the random sampling methods employed? If not, then still, the mechanism by which the participants were selected to be included in the study needs to be clarified, including the proportion included in each study setting.

[5] Inclusion and exclusion criteria (page 7): The exclusion criteria normally apply to those who already met the inclusion criteria, which wasn't the case in this manuscript, so once the inclusion criteria applied, none of the exclusion criteria excluded any participants.

[b] Inclusion criterion (a) “…a language interpreter was needed.” Why? In the results section, table 1 indicates 13 intermediate, 1 advanced, and 3 fluent speakers. Does this not conflict with inclusion criterion a? In Table 2, only beginners and not at all are presented. This needs to be sorted out and clarified.

[C] The exclusion criterion (c) seems to state informed consent. But on page 10, you stated that “participants received written information and provided written consent prior to enrolment.”

Discussion

[6] The discussion lacks depth and supporting references.

[7] The "Conclusion" section is missing.
